# Supplementary material for: Evaluating the feasibility, adoption, cost-effectiveness, and sustainability of telemedicine interventions in managing COVID-19 within low-and-middle-income countries (LMICs): A systematic review
Source: PLOS Digit Health. 2025 Apr 8;4(4):e0000771. doi: 10.1371/journal.pdig.0000771 (PMC11978082; doi:10.1371/journal.pdig.0000771)
Supplement: S1 Appendix — (DOCX) [file pdig.0000771.s001.docx]

**S1 Appendix. Search Strategy.** Systematic review search strategy used across the following 6 Public Health and Health Science Databases/Registries: PubMed, CINAHL, Web of Science, Embase, Global Health, and Cochrane Library.

| Telemedicine  (telemedicine OR mhealth OR mobile health OR eHealth OR telehealth |
| --- |
| COVID-19  AND coronavirus OR covid-19 OR SARS-COV-2) |
| Implementation Science Outcomes  AND cost OR economic* OR feasib* OR acceptance OR acceptab* OR adopt* OR uptake OR “utility” OR “practical*” OR appropriate* OR fidelity OR coverage OR “effectiveness” OR ”implement*” OR “outcomes” OR "Costs and Cost Analysis"[Mesh] OR "feasibility studies"[Mesh]) |
| LMICs  AND ("developing countries"[tw] OR "developing country"[tw] OR "developing economy"[tw] OR "developing economies"[tw] OR "lmic"[tw] OR "lmics"[tw] OR "low income countries"[tw] OR "low income country"[tw] OR "low income economies"[tw] OR "low income economy"[tw] OR "middle income countries"[tw] OR "middle income country"[tw] OR "middle income economies"[tw] OR "middle income economy"[tw] OR "third world country"[tw] OR "third world countries"[tw] OR "global south"[tw] OR "central asia"[tw] OR "western asia"[tw] OR "southeastern asia"[tw] OR "indian ocean islands"[tw] OR "central america"[tw] OR "south america"[tw] OR "latin america"[tw] OR "eastern europe"[tw] OR "transcaucasia"[tw] OR "caribbean region"[tw] OR "africa"[tw] OR "caribbean"[tw] OR "pacific islands"[tw] OR "afghan"[tw] OR "afghanistan"[tw] OR "albania"[tw] OR "albanian"[tw] OR "algeria"[tw] OR "algerian"[tw] OR "angola"[tw] OR "angolan"[tw] OR "antigua"[tw] OR "antigua and barbuda"[tw] OR "argentina"[tw] OR "argentinian"[tw] OR "armenia"[tw] OR "armenian"[tw] OR "azerbaijan"[tw] OR "azerbaijani"[tw] OR "bajan"[tw] OR "bangladesh"[tw] OR "bangladeshi"[tw] OR "barbadian chile"[tw] OR "barbados"[tw] OR "barbuda"[tw] OR "belarus"[tw] OR "belarusian"[tw] OR "belize"[tw] OR "belizean"[tw] OR "benin"[tw] OR "beninese"[tw] OR "bhutan"[tw] OR "bhutanese"[tw] OR "bolivia"[tw] OR "bolivian"[tw] OR "bosnia"[tw] OR "bosnian"[tw] OR "botswana"[tw] OR "brazil"[tw] OR "brazilian"[tw] OR "bulgaria"[tw] OR "bulgarian"[tw] OR "burkina faso"[tw] OR "burkinabes"[tw] OR "burmese"[tw] OR "burundi"[tw] OR "burundian"[tw] OR "cambodia"[tw] OR "cambodian"[tw] OR "cameroon"[tw] OR "cameroonian"[tw] OR "cape verdan"[tw] OR "cape verde"[tw] OR "cape verdeans"[tw] OR "central african"[tw] OR "central african republic"[tw] OR "chad"[tw] OR "chadian"[tw] OR "chilean"[tw] OR "china"[tw] OR "chinese"[tw] OR "colombia"[tw] OR "colombian"[tw] OR "comorian"[tw] OR "comoros"[tw] OR "congo"[tw] OR "congolese"[tw] OR "costa rica"[tw] OR "costa rican"[tw] OR "cote d'ivoire"[tw] OR "cuba"[tw] OR "cuban"[tw] OR "djibouti"[tw] OR "dominica"[tw] OR "dominican"[tw] OR "east timor"[tw] OR "east timor eye"[tw] OR "ecuador"[tw] OR "ecuadorian"[tw] OR "egypt"[tw] OR "egyptian"[tw] OR "el salvador"[tw] OR "eritrea"[tw] OR "eritrean"[tw] OR "ethiopia"[tw] OR "ethiopian"[tw] OR "ezswatini"[tw] OR "fiji"[tw] OR "fijian"[tw] OR "filipino"[tw] OR "gabon"[tw] OR "gabonese"[tw] OR "gambia"[tw] OR "gambian"[tw] OR "gaza"[tw] OR "georgia"[tw] OR "georgian"[tw] OR "ghana"[tw] OR "ghanaian"[tw] OR "grenada"[tw] OR "grenadian"[tw] OR "guatemala"[tw] OR "guatemalan"[tw] OR "guinea"[tw] OR "guinean"[tw] OR "guyana"[tw] OR "guyanese"[tw] OR "haiti"[tw] OR "haitian"[tw] OR "honduran"[tw] OR "honduras"[tw] OR "india"[tw] OR "indian"[tw] OR "indonesia"[tw] OR "indonesian"[tw] OR "iran"[tw] OR "iranian"[tw] OR "iraq"[tw] OR "iraqi"[tw] OR "ivory coast"[tw] OR "jamaica"[tw] OR "jamaican"[tw] OR "jordan"[tw] OR "jordanian"[tw] OR "kazakhstan"[tw] OR "kenya"[tw] OR "kenyan"[tw] OR "kiribati"[tw] OR "north korea"[tw] OR "north korean"[tw] OR "kosovar"[tw] OR "kosovo"[tw] OR "kyrgyz"[tw] OR "kyrgyzstan"[tw] OR "Lao"[tw] OR "laos"[tw] OR "laotian"[tw] OR "lebanese"[tw] OR "lebanon"[tw] OR "lesotho"[tw] OR "liberia"[tw] OR "liberian"[tw] OR "libya"[tw] OR "libyan"[tw] OR "lithuania"[tw] OR "lithuanian"[tw] OR "macedonia"[tw] OR "macedonian"[tw] OR "madagascar"[tw] OR "malagasy"[tw] OR "malawi"[tw] OR "malawian"[tw] OR "malaysia"[tw] OR "malaysian"[tw] OR "maldives"[tw] OR "maldivian"[tw] OR "mali"[tw] OR "malian"[tw] OR "marshall islands"[tw] OR "marshallese"[tw] OR "mauritania"[tw] OR "mauritanian"[tw] OR "mauritian"[tw] OR "mauritius"[tw] OR "mexican"[tw] OR "mexico"[tw] OR "micronesia"[tw] OR "micronesian"[tw] OR "moldova"[tw] OR "moldovan"[tw] OR "mongolia"[tw] OR "mongolian"[tw] OR "montenegrin"[tw] OR "montenegro"[tw] OR "moroccan"[tw] OR "morocco"[tw] OR "mozambican"[tw] OR "mozambique"[tw] OR "myanmar"[tw] OR "namibia"[tw] OR "namibian"[tw] OR "nepal"[tw] OR "nepalese"[tw] OR "nicaragua"[tw] OR "nicaraguan"[tw] OR "niger"[tw] OR "nigeria"[tw] OR "nigerian"[tw] OR "pakistan"[tw] OR "pakistani"[tw] OR "palau"[tw] OR "palauan"[tw] OR "panama"[tw] OR "panamanian"[tw] OR "papua new guinea"[tw] OR "papua new guinean"[tw] OR "paraguay"[tw] OR "paraguayan"[tw] OR "peru"[tw] OR "peruvian"[tw] OR "philippines"[tw] OR "principe"[tw] OR "romania"[tw] OR "romanian"[tw] OR "russia"[tw] OR "russian"[tw] OR "rwanda"[tw] OR "rwandan"[tw] OR "saint kitts"[tw] OR "saint lucia"[tw] OR "saint vincent"[tw] OR "salvadoran"[tw] OR "samoa"[tw] OR "samoan"[tw] OR "santomea"[tw] OR "sao tome"[tw] OR "senegal"[tw] OR "senegalese"[tw] OR "serbia"[tw] OR "serbian"[tw] OR "seychelles"[tw] OR "seychellois"[tw] OR "sierra leone"[tw] OR "sierra leoneans"[tw] OR "solomon islander"[tw] OR "solomon islands"[tw] OR "somalia"[tw] OR "somalian"[tw] OR "south africa"[tw] OR "south african"[tw] OR "sri lanka"[tw] OR "sri lankan"[tw] OR "sudan"[tw] OR "sudanese"[tw] OR "suriname"[tw] OR "swazi"[tw] OR "swaziland"[tw] OR "syria"[tw] OR "syrian"[tw] OR "tadzhik"[tw] OR "tajik"[tw] OR "tajikistan"[tw] OR "tanzania"[tw] OR "tanzanian"[tw] OR "thai"[tw] OR "thailand"[tw] OR "togo"[tw] OR "togolese"[tw] OR "tonga"[tw] OR "tongan"[tw] OR "tunisia"[tw] OR "tunisian"[tw] OR "turkey"[tw] OR "turkish"[tw] OR "turkmen"[tw] OR "turkmenistan"[tw] OR "tuvalu"[tw] OR "tuvaluans"[tw] OR "uganda"[tw] OR "ugandan"[tw] OR "ukraine"[tw] OR "ukrainian"[tw] OR "uzbek"[tw] OR "uzbekistan"[tw] OR "vanuatu"[tw] OR "venezuela"[tw] OR "venezuelan"[tw] OR "vietnam"[tw] OR "vietnamese"[tw] OR "west bank"[tw] OR "yemen"[tw] OR "yemeni"[tw] OR "yemenite"[tw] OR "zambia"[tw] OR "zambian"[tw] OR "zimbabwe"[tw] OR "zimbabwean"[tw] |
| RCTs  AND randomized controlled trial[pt] OR controlled clinical trial[pt] OR randomized[tiab] OR placebo[tiab] OR drug therapy[sh] OR randomly[tiab] OR trial[tiab] OR groups[tiab] |
| Restrictions are no reviews and non-human studies  NOT animals [mh] NOT humans [mh]) OR “review”[pt]"Treatment Adherence and Compliance"[Mesh])) |
