# Supplementary material for: Evaluating the feasibility, adoption, cost-effectiveness, and sustainability of telemedicine interventions in managing COVID-19 within low-and-middle-income countries (LMICs): A systematic review
Source: PLOS Digit Health. 2025 Apr 8;4(4):e0000771. doi: 10.1371/journal.pdig.0000771 (PMC11978082; doi:10.1371/journal.pdig.0000771)
Supplement: Attachment — Submitted filename: Response_to_Reviewers_auresp_2.docx [file pdig.0000771.s005.docx]

PLOS Digital Health Journal

Submission Date: December 7, 2024

Dear Editor,

Thank you for the opportunity to submit a revised version of our research article titled, “Evaluating Feasibility, Adoption, Cost-effectiveness, and Sustainability of Telemedicine Interventions in Managing COVID-19 within Low-and-Middle-Income Countries (LMICs): A Systematic Review,” for publication to PLOS Digital Health. Our team expresses sincere gratitude to the reviewers for their invaluable feedback, which has significantly enriched the quality of our manuscript. We have diligently incorporated all the suggestions provided by the reviewers, ensuring that every concern raised during the review process has been meticulously addressed in the revised article.

With this resubmission, we reaffirm our commitment to contributing to the scholarly discourse on telemedicine interventions amidst the challenges posed by the COVID-19 pandemic in LMICs. Alongside this letter, you will find the requisite documents accompanying our submission. We eagerly anticipate the opportunity for our work to be considered for publication in *PLOS* Digital Health.

- A marked-up copy of the manuscript that highlights changes made to the original version: labeled ‘Revised Manuscript with Track Changes’.
- An unmarked version of the revised manuscript without tracked changes: labeled ‘Manuscript’.

Below are point-by-point remarks describing how we addressed the concerns of the reviewers.

**Reviewer 1 Comment:** *Thank you for your hard work addressing the suggested revisions. The paper is in great shape, with limitations appropriately acknowledged.*

**Author Response**: Thank you for your thorough review and insightful feedack.

**Reviewer 3 Comment 1:** *Evaluating feasibility, adoption, cost-effectiveness, and sustainability of telemedicine interventions in managing COVID-19 within low- and middle-income countries (LMICs): A Systematic Review.*

*1. Given the importance of implementing evidence-based interventions for pandemics such as COVID-19 and their urgency in low-resource settings, the paper examines the feasibility, adoption, cost-effectiveness, and sustainability of telemedicine interventions. While a growing literature has addressed both the use of telemedicine and the urgency of implementing proven interventions for pandemic, this paper is one of the few that examines the implementation outcomes of such interventions. Attaining implementation outcomes is important for two reasons: (1) we have little data about the telemedicine’s feasibility, adoption, cost-effectiveness, and sustainability; and (2) ensuring that those outcomes are attained is critical for subsequent tests of telemedicine’s clinical effectiveness (one cannot conclude that telemedicine is effective without ensuring that it was implemented well). While claiming the importance of attaining implementation outcomes is not novel, direct examination of their implementation via the lens of implementation outcomes is under-studied. Relevant literature is succinctly and adequately reported.*

**Author Response 1**: Thank you for acknowledging the significance and novelty of our study. We agree that evaluating the feasibility, adoption, cost-effectiveness, and sustainability of telemedicine interventions in low-resource settings is crucial for effective pandemic response and management. We appreciate your recognition of the need for comprehensive data on telemedicine’s implementation outcomes. Our aim was to address this gap in the literature to support telemedicine's overall effectiveness. We are pleased that you found our literature review succinct and adequately reported. We hope these revisions meet your expectations and further enhance our manuscript.

**Reviewer 3 Comment 2:**

*2. The manuscript would be strengthened by a list of the (clearly stated or enumerated) research aims, questions, or hypotheses that the systematic review aims to inform.*

*Methods for the systematic review are described clearly, including the search strategy, inclusion and exclusion criteria, and data extraction methods.*

**Author Response 2**: Thank you for this valuable suggestion. We have revised the abstract to include an outline of the research aims of the systematic review. This section now clearly enumerated the specific goals of the review, including the feasibility, adoption, cost-effectiveness, and sustainability of telemedicine interventions for COVID-19 management in LMICs.

**Reviewer 3 Comment 3:**

*3. Moreover, the authors conducted a bias risk assessment using the Mixed methods Appraisal Tool. The scoring method and interpretation of colors for cells in Table 2 need better explanation.*

**Author Response 3**: Thank you for highlighting this area that needs improvement. We have updated the risk bias section of the manuscript to provide a more detailed explanation of the scoring method and the interpretation of cell colors for Table 3. We clarified that the Mixed Methods Appraisal Tool assigns scores to which studies meet the criteria, with higher scores indicating a lower risk of bias.

**Reviewer 3 Comment 4:**

*4. Likewise, this reviewer requests more detail about how each of the implementation outcomes was measured in each study. Were standardized measures employed? Who reported on implementation outcome attainment? What data sources? How harmonized was measurement of a given outcome across studies?*

**Author Response 4**: Thank you for your assessment requesting further clarification on the measurement of each implementation outcomes in each study. In addition to the information provided within Table 2, which outlines the implementation research outcome, further information was provided within the Quality of Evidence section of the manuscript.

**Reviewer 3 Comment 5:**

*5. Table 3 reports the service outcomes addressed in each study, but the literature review and manuscript framing focus primarily on implementation outcomes. Including the service outcomes increases the manuscript’s value, but more discussion is needed. Why were these outcomes included in data reporting? Did any of the three papers address inter-relationships among implementation outcomes, or any analysis of association between implementation and service outcomes?*

**Author Response 5**: Thank you for the feedback on providing context within the evaluation of the service outcomes. Additional information regarding service outcomes were added to the Results section of the paper and further discussion around their categorization was added to the Quality of Evidence section.

**Reviewer 3 Comment 6:**

*6. In the same vein, the authors report available effectiveness data. This reviewer requests that the manuscript frame the aims/ questions/ hypotheses driving the systematic review before the methods section and include service outcomes and effectiveness outcomes in those aims.*

*•	the manuscript warrant such treatment?*

*•	If the paper is considered unsuitable for publication in its present form, does the study itself show sufficient potential that the authors should be encouraged to resubmit a revised version?*

**Author Response 6:**

Thank you for the feedback on restructuring the manuscript to include details on the aims/questions prior to the methods section. We have included the aims and questions in the introduction section, including the service and effectiveness outcomes in the aims. We will be including this in the revised version of the manuscript as the study still shows the effectiveness of telemedicine interventions in LMICs.

**Reviewer 3 Comment 7:**

*7. This manuscript’s methods and findings, albeit limited as the authors acknowledge, are important additions to the scant evidence about the topic. However more detail as noted above will strengthen the paper’s contributions.*

**Author Response 7:** Thank you for recognizing the value of our methods and findings despite their acknowledged limitations. We agree that the additional details requested will further strengthen the manuscript's contributions. We have included these enhancements in our revisions to provide a more comprehensive analysis. We appreciate the time and effort you have dedicated to improving our paper and look forward to the final evaluation.

All authors have read and agree to the submission of the revised manuscript. Along with my co-authors, I wish to thank each of you for your insights. We hope that the changes made to the manuscript are suitable for resubmission in *PLOS Digital Health*. We look forward to hearing from you.

Sincerely,

Emmanuel Peprah, PHD

NYU School of Global Public Health

Department of Global and Environmental Health
