# Supplementary material for: Evaluating the feasibility, adoption, cost-effectiveness, and sustainability of telemedicine interventions in managing COVID-19 within low-and-middle-income countries (LMICs): A systematic review
Source: PLOS Digit Health. 2025 Apr 8;4(4):e0000771. doi: 10.1371/journal.pdig.0000771 (PMC11978082; doi:10.1371/journal.pdig.0000771)
Supplement: Attachment — Submitted filename: Response to Reviewers.docx [file pdig.0000771.s004.docx]

PLOS Digital Health Journal

Submission Date: June 18, 2024

Dear Editor,

We are delighted to present a refined version of our research article titled, “Evaluating Feasibility, Adoption, Cost-effectiveness, and Sustainability of Telemedicine Interventions in Managing COVID-19 within Low-and-Middle-Income Countries (LMICs): A Systematic Review,” for your esteemed consideration at PLOS Digital Health. Our team expresses sincere gratitude to the reviewers for their invaluable feedback, which has significantly enriched the quality of our manuscript. We have diligently incorporated all the suggestions provided by the reviewers, ensuring that every concern raised during the review process has been meticulously addressed in the revised article.

With this resubmission, we reaffirm our commitment to contributing to the scholarly discourse on telemedicine interventions amidst the challenges posed by the COVID-19 pandemic in LMICs. Alongside this letter, you will find the requisite documents accompanying our submission. We eagerly anticipate the opportunity for our work to be considered for publication in *PLOS* Digital Health.

- A marked-up copy of the manuscript that highlights changes made to the original version: labeled ‘Revised Manuscript with Track Changes’.
- An unmarked version of the revised manuscript without tracked changes: labeled ‘Manuscript’.

Below are point-by-point remarks describing how we addressed the concerns of the reviewers.

**Reviewer 1 Comments** **1.** *The authors in the introduction describe telehealth as “seamless” or “critical.” I do not think there is enough evidence to support such strong language. Please temper the introduction considerably. Telehealth is by no means a panacea.*

**Author Response 1**: We concur with the reviewer's insightful comment and acknowledge the dynamic nature of telemedicine opportunities, which are contingent upon the unique needs of diverse populations and health systems. In response, we have refined the language in the Introduction section to underscore the potential outcomes that may vary depending on the resources available within different contexts. This adjustment aims to accurately portray the nuanced landscape of telemedicine implementation across various settings.

**Reviewer 1 Comments** **2.** *In the methods, the authors write that “Subsequently, in (November 2023), we refined our inquiry by delving into public health and health science databases, enabling us to gain valuable insights into our target population, intervention, and outcome variables.” What prompted this change over a year later?*

**Author Response 2**: We thank the reviewer for a thorough assessment of our manuscript. The preliminary literature review and assessment for this manuscript was conducted in October 2022 to aid in authoring the first draft of our paper. Between October 2022 and November 2023, the manuscript underwent various revisions following several rounds of review conducted by the contributors of this paper. During the final draft of the manuscript, we performed a second search employing our original search strategy to ensure that no additional articles published during the authoring period had been missed. This process adheres to guidelines for systematic reviews. Furthermore, given the surge in telemedicine research efforts and rapidly evolving findings for COVID-19 over the recent years, subsequent searches were needed to allow our team to incorporate new literature that met our inclusion criteria. We updated the Methods section to reflect these dates more clearly and have also included an Appendix to further provide our search strategy for the various databases utilized for our systematic review.

**Reviewer 1 Comments** **3.** *The authors make a good point that the three studies included – from Brazil, Turkey, and China – are from MICs. The authors should dedicate perhaps a paragraph or two to studies looking at telemedicine LMICs. Perhaps they can describe some of the papers that were selected out – might it be that the low representation of studies from LMICs is due to the lower feasibility of conducting telemedicine studies that would have been included in this meta-analysis given LMIC resource constraints? Taken a step further, this paper seeks to explore telemedicine in LMICs but seems to ‘select out’ papers from LMIC settings, in a way, shooting itself in the foot. I do not think the authors need to revise their methods at all. They just need to perhaps highlight this in the limitations if they agree with the above.*

**Author Response 3**: We extend our gratitude to the reviewer for providing valuable feedback. In our original manuscript's Discussion section, we openly acknowledged the dearth of peer-reviewed articles focusing explicitly on telemedicine and its implementation outcomes concerning COVID-19 in LMICs. Our search strategy was meticulously crafted to encompass low, low-middle, middle, and upper-middle income countries as classified by the World Bank, aligning with the criteria outlined (Appendix A in S1 File). The limited number of articles identified through our comprehensive search and meeting our stringent inclusion criteria underscores the novelty of both the COVID-19 pandemic and its associated publications during this period.

To offer further elucidation on this observed scarcity, we augmented the manuscript with additional background information detailing the hurdles to telemedicine implementation within low-income countries, particularly in the introduction section. Furthermore, we provided insights into the reasons behind the paucity of available articles linking COVID-19 and telemedicine within the context of LMIC settings, as outlined in the limitations section of the manuscript.

**Reviewer 1 Comments** **4.** *The authors could include in the discussion 1) a paragraph on next steps – what are actionable findings from their results? And 2) a section on telehealth in LIC and LMIC settings.*

**Author Response 4**: We express our gratitude to the reviewer for providing invaluable feedback, which has prompted us to incorporate a dedicated paragraph on actionable findings in the Discussion section. Additionally, we have meticulously revised the Introduction section to accentuate the significance of telehealth in Low-Income Countries (LICs). We are confident that these enhancements effectively address the concerns raised by this reviewer.

**Reviewer 1 Comments** **5.** *It is interesting to note that all the authors and affiliations are in HICs. I wonder if this too is a limitation that should be addressed, especially if there is a sincere effort to “decolonize” global health research in the literature.*

**Response 5**: We thank the reviewer for their valuable feedback and recognize the imperative to enhance representation of researchers from LMICs. It is essential to emphasize that while the systematic review was commissioned at a HIC institution, the senior author and many co-authors of this manuscript hail from LMIC backgrounds. Our authorship team comprises individuals from both HICs and LMICs, acknowledging that our diverse perspectives may be shaped by the composition of our team, as is the case with any research endeavor.

**Reviewer 2 Comment 1:** *Suggest that the authors reconsider solely using the "taxonomy of implementation outcomes defined by Proctor et al. -- for feasibility, adoption, cost effectiveness and sustainability (of telemedicine) in this paper. A review of these concepts as these are used in the eHealth / digital health space must be evaluated, adopted for the study...*

*Specifically: the authors wanted to investigate the adoption and sustainability of telemedicine --- the authors did not establish these 2 attributes of telemedicine -- and thus a better understanding of these concepts can enhance the authors' discourse*

**Author Response 1:** The reviewer’s insightful comment has improved our manuscript, which has prompted us to delve deeper into the assessment of implementation outcomes outlined in Table 1. In response, we have undertaken significant efforts to enrich our systematic analysis by not only scrutinizing the results but also meticulously listing them in Table 3 and relevant sections throughout the manuscript.

Our findings reveal a nuanced landscape concerning the adoption and sustainability of telemedicine interventions in LMICs, as depicted by the current literature. While we have encountered anecdotal evidence suggesting the existence of interventions employed in LMICs, it is noteworthy that the literature does not adequately capture the diversity of experiences among implementers. This disparity may stem from a myriad of factors, including a lack of comprehensive understanding of these concepts within the telehealth community and among implementers and researchers in LMICs, compounded by the relatively nascent nature of implementation research in this field.

Against this backdrop, we firmly believe that our manuscript holds the potential to serve as a guiding beacon for implementers seeking to navigate the intricate terrain of telemedicine interventions within LMIC settings. By elucidating the key challenges and potential avenues for improvement, we aim to empower implementers to develop manuscripts that shed light on their specific experiences in implementing telehealth for COVID-19. We envision that the representative publications highlighted within our manuscript will not only offer valuable insights but also inspire others to contribute their unique perspectives and lessons learned from similar endeavors. Through collective efforts and knowledge-sharing, we aspire to foster a robust and inclusive discourse surrounding telemedicine implementation in LMICs, ultimately driving positive impact and advancing the field towards greater efficacy and sustainability.

All authors have read and agree to the submission of the revised manuscript. Along with my co-authors, I wish to thank each of you for your insights. We hope that the changes made to the manuscript are suitable for resubmission in *PLOS Digital Health*. We look forward to hearing from you.

Sincerely,

Emmanuel Peprah, PHD

NYU School of Global Public Health

Department of Global and Environmental Health
